# 571. A Report of the Postmarketing Spontaneous Safety Data over 24 Years for GSK’s Measles-Mumps-Rubella (MMR) Vaccine

**DOI:** 10.1093/ofid/ofac492.624

**Published:** 2022-12-15

**Authors:** Tina Singh, Giacomo Casabona, Remon Abu-Elyazeed, Volker Vetter, Fanny Hergibo

**Affiliations:** GSK, Wavre, Brabant Wallon, Belgium; GSK, Wavre, Brabant Wallon, Belgium; GSK, Philadelphia, PA, USA; GSK, Munich, Germany; GSK, Wavre, Brabant Wallon, Belgium

## Abstract

**Background:**

GSK’s measles-mumps-rubella (MMR) vaccine is indicated for active immunization of individuals aged 12 months and older against these 3 serious illnesses. It is licensed in > 100 countries worldwide not including the US. In clinical trials, it elicited robust immune responses in children and was well tolerated. Monitoring the vaccine’s real-world safety profile in the general population continues, and rapid collection of data on its real-world use is a strength of passive safety surveillance. We provide an overview of the postmarketing spontaneous safety data over 24 years for GSK’s MMR vaccine.

**Methods:**

Spontaneous adverse events (AE) reports following vaccination with GSK’s MMR vaccine were collected from GSK’s Global Safety Database from 4-December-1997 until 1-March-2022. Annual reporting trends for pyrexia, rashes (commonly reported in the pediatric population), and events of clinical interest like febrile convulsions and neurological conditions were analyzed from 1998 until 2021. The patient exposure was estimated by the number of doses distributed (DD).

**Results:**

Over the reviewed period, ∼445 million doses of GSK’s MMR vaccine were distributed worldwide, and 29,504 cases were spontaneously reported (rate: 6.63 cases reported/100,000 DD). Of all spontaneous AE reports, 9,960 (33.76%) were considered as serious. For the period analyzed, pyrexia and rash were the most frequently reported AEs following vaccination with GSK’s MMR vaccine (Table). Reports of febrile convulsions had a mean frequency of 0.27 cases/100,000 DD (Table); reports of neurological conditions (*encephalitis* and/or *panencephalitis* and/or *meningitis* and/or *meningism*) had a mean frequency of 0.07 cases/100,000 DD (Table) and did not show a trend of increased incidence over time.

**Figure:**
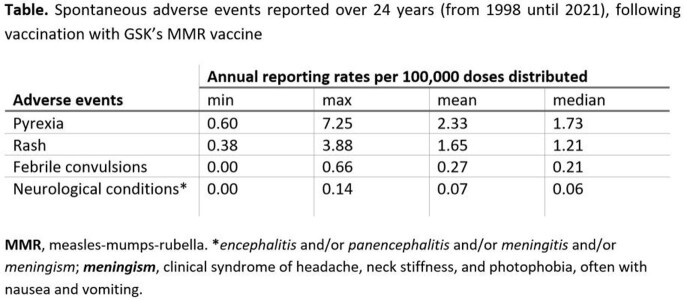

**Conclusion:**

The postmarketing safety data analyzed showed that the safety profile of GSK’s MMR vaccine is consistent with that observed in clinical trials and remains supportive of its overall safety profile in routine use. GSK continues monitoring the safety of its MMR vaccine.

**Funding:**

GlaxoSmithKline Biologicals SA

**Acknowledgment:** Adina Truta/Julie Mellery provided medical writing/editorial support (Modis c/o GSK)

**Disclosures:**

**Tina Singh, MD physician**, GSK: Employee of the GSK group of companies and having restricted GSK shares in the GSK group of companies **Giacomo Casabona, MD**, GSK: I’m an employee of the GSK group of companies and shareholder in the GSK group of companies **Remon Abu-Elyazeed, MD, PhD**, GSK: Employee of the GSK group of companies and having GSK stocks in the GSK group of companies **Volker Vetter, MD**, GSK: I’m an employee of the GSK group of companies and hold shares in the GSK group of companies **Fanny Hergibo, Pharm.D.**, GSK: Employee of the GSK group of companies.

