# Correction to: 571. A Report of the Postmarketing Spontaneous Safety Data over 24 Years for GSK’s Measles-Mumps-Rubella (MMR) Vaccine

**DOI:** 10.1093/ofid/ofad249

**Published:** 2023-05-08

In the original publication of this abstract, [Singh T, Casabona G, Abu-Elyazeed R, et al. A Report of the Postmarketing Spontaneous Safety Data over 24 Years for GSK’s Measles-Mumps-Rubella (MMR) Vaccine, Open Forum Infectious Diseases, Volume 9, Issue Supplement_2, ofac492.624, https://doi.org/10.1093/ofid/ofac492.624], there were additional GSK locations for members of the byline which were not recorded as affiliations. This has been corrected in the version of the proof currently available online.

